# From Functional Genomics to Functional Immunomics: New Challenges, Old Problems, Big Rewards 

**DOI:** 10.1371/journal.pcbi.0020081

**Published:** 2006-07-28

**Authors:** Ulisses M Braga-Neto, Ernesto T. A Marques

**Affiliations:** Heinrich Heine University, Germany

## Abstract

The development of DNA microarray technology a decade ago led to the establishment of functional genomics as one of the most active and successful scientific disciplines today. With the ongoing development of immunomic microarray technology—a spatially addressable, large-scale technology for measurement of specific immunological response—the new challenge of functional immunomics is emerging, which bears similarities to but is also significantly different from functional genomics. Immunonic data has been successfully used to identify biological markers involved in autoimmune diseases, allergies, viral infections such as human immunodeficiency virus (HIV), influenza, diabetes, and responses to cancer vaccines. This review intends to provide a coherent vision of this nascent scientific field, and speculate on future research directions. We discuss at some length issues such as epitope prediction, immunomic microarray technology and its applications, and computation and statistical challenges related to functional immunomics. Based on the recent discovery of regulation mechanisms in T cell responses, we envision the use of immunomic microarrays as a tool for advances in systems biology of cellular immune responses, by means of immunomic regulatory network models.

## Introduction

During the past decade, the highly successful field of functional genomics experienced huge growth as a result of the development of DNA microarray technology [1–4], which made it possible for the first time to measure the RNA expression of thousands of genes in parallel, in a single assay. Immune responses are complex phenomena that supervene on genomics, that is, immune responses ultimately depend on the expression of genes inside a variety of cells, but explaining the function of the immune system only in terms of gene expression in those cells would constitute a reductionist approach. While studying the immune system in terms of genomics is an important goal [5,6], the function of the immune system, from antigen processing to epitope-specific immune responses, may be better understood through an integrated approach that takes into account properties of the immune system as a whole.

We quote from [7], “The immunome is the detailed map of immune reactions of a given host interacting with a foreign antigen, and immunomics is the study of immunomes.” Whereas functional genomics strives to identify the role of genes in cellular processes via the paradigm of hybridization of mRNA to complementary DNA, functional immunomics aims to identify the roles of chemical/biological targets involved in immunological processes via the paradigm of specific cellular and humoral immune responses elicited by antigens presented to the immune system [8–11]. This is an effort that promises great rewards, both in terms of our basic understanding of the immune system and in terms of disease diagnosis/prognosis [12] and the design of vaccines [13–15] to combat a variety of human infirmities ranging from pathogenic infections to allergies and cancer.

### 

#### Enabling technologies.

Functional genomics was made possible by the significant advances that had previously been made in sequential genomics, including not only the massive efforts required to identify genome-wide DNA sequences [16], but also the computational methods used to parse and align those sequences [17]. Sequential genomic data are deposited in large public-access databanks such as GenBank [18], and researchers or companies who make DNA microarrays use the sequences in these databases as probes. In a similar fashion, the field of functional immunomics has now come of age as a result of advances in sequential immunomics, which consists of methods to catalogue the chemical/biological targets capable of eliciting an immune response, also known as *epitopes*. Computational and statistical methods are now available for automated large-scale epitope prediction (please see the next subsection), in addition to classical immunoassays such as ELISPOT [19] and tetramer staining by flow cytometry [20] that together enable high throughput identification of epitopes. Recently, a coordinated effort has been initiated by the National Institute of Allergy and Infectious Disease at the US National Institutes of Health, under the auspices of the Large-Scale Antibody and T Cell Epitope Discovery Program (in which the authors of this paper participate), to create an integrated immunome database and resources such as a toolbox of epitope prediction methods. This initiative is designed to identify immune epitopes from selected infectious agents; the information will be made freely available to scientists worldwide through the Immune Epitope Database and Analysis Resource (IEDB) [7,21,22] (http://www.immuneepitope.org). The sequencing information produced by this and other epitope mapping efforts being carried out will be essential for the construction of immunomic microarrays (discussed in detail below), leading to an experimental paradigm similar to that employed in functional genomics.

#### Computational epitope prediction methods.

The immune system recognizes antigen via binding of antibody (humoral response) or T cell receptors (cellular response) to self or foreign proteins. B cell epitopes correspond in general to the 3-D features on the surface of antigen where recognition by the immune system occurs; a continuous or linear epitope is a sequential fragment from the protein sequence, while a discontinuous or conformational epitope is composed of several fragments scattered along the protein sequence and brought together in spatial proximity when the protein is folded [23]. Humoral response is targeted mainly at conformational epitopes, which may represent up to 90% of the total B cell responses. This makes prediction of B cell epitopes a hard problem [24], even more so because B cell responses are virtually only restricted by immunoglobulin access to the epitope, B cell receptor activation, and self versus no-self discrimination rules of the immune system. Ideally B cell prediction systems would use 3-D surface models of the protein antigens and measure surface energy interactions of variable regions of the immunoglobulins that correlate with B cell activation. However, so far B cell prediction systems make estimations of the probability of a primary peptide sequence being present at the surface of a protein based on hydrophilicity and secondary structures [25]. Cellular responses, on the other hand, are restricted through the binding of T cell receptors to short linear peptides, which are bound by a specific groove in two main classes of major histocompatibility complex (MHC) molecules, and presented on the surface of cells to the T cell receptors of CD4^+^ and CD8^+^ cells [26]. Binding affinity between the peptide and the MHC molecule is therefore a necessary requirement for effective cellular immune response. A complicating factor is the highly polymorphic nature of the MHC molecule, which displays large variability in human populations. Using experimental affinity data deposited in public databases as training data, researchers have developed statistical methods to predict the MHC affinity of a given unknown peptide [27–42]. Typically, such computational epitope prediction methods scan the full length of pathogen or self-immunogenic protein sequences by taking consecutive overlapping peptides. In addition, such methods can predict “promiscuous” epitopes, that is, the ones that bind to a large class of different MHC alleles, know as supertypes [43]. Computational MHC-binding prediction methods have become essential for the systematic search for epitopes, in situations where techniques such as ELISPOT and flow cytometry are effectively impractical due to the large number of peptides to be assayed.

Early MHC-binding studies identified characteristic amphipathic chemical patterns on the binding peptides [44], and enhanced versions of these systems continue in use in association with other methods [41,44,45]. Today, the most basic MHC-binding prediction methods are based on the identification of specific amino acids commonly found at particular positions, called binding motifs, within peptides that bind to a specific MHC molecule. However, all the amino acids of a peptide bound to an MHC groove (normally 8–10 amino acids for MHC I and 8–14 amino acids for MHC II) can potentially play a positive or negative role in binding, and more complex methods assign positive or negative values for each amino acid at each position of a peptide and combine these values to define scores that predict binding; these “quantitative-matrix” approaches have been very successful. One of the limitations of the “quantitative-matrix” approach is that it does not take into consideration the influences of interactions between amino acids at different peptide positions of the epitope. The value of these interactions are difficult to measure and have been explored only in a limited fashion by combining pair-wise interactions between two peptide positions [46]. The combination of independent binding calculated by quantitative matrices with coefficients derived from the pair-wise interaction provided better predictions. Moreover, quantitative-matrix scores have been generated for several HLA alleles, and studies using HLA sequence homology have allowed the development of virtual quantitative matrices to be applicable to many more HLA alleles [28,39]. General methods for MHC-binding prediction systems, such as artificial neural networks, and statistical models such as Hidden Markov, can incorporate nonlinear complex interactions between the MHC molecule and the peptide epitope, and can evolve as more data is included in the training set. These general methods have shown to be potentially superior to the previous ones [27,37]. The greatest challenge, however, of the general methods is that, to be reliable, they require a larger amount of peptide-binding data. Strategies using query-by-committee approaches to compare predictions from different training sets have been used to identify the most informative peptide-binding data to be determined in the biochemical binding assays in order to more efficiently build a representative training dataset [40]. Recently a collection of more than 48,000 peptide binding affinities of class I molecules were made public [47]. The development of prediction models will be greatly accelerated by this community resource benchmark. We remark that epitope recognition by the immune system involves more than a receptor–ligand problem between the MHC molecule and a peptide, as other biological processes are involved in preparation of the peptide for loading into the MHC molecule. Epitope prediction systems continue to evolve, and many steps in antigen processing and presentation required for development of cognate immune responses are being modeled and combined into rational prediction systems. The greatest current challenge is the development of models incorporating the rules of B cell and T cell receptor engagement and their possible outcomes.

#### The growth “boom” of immunomics.

**Figure 1:**
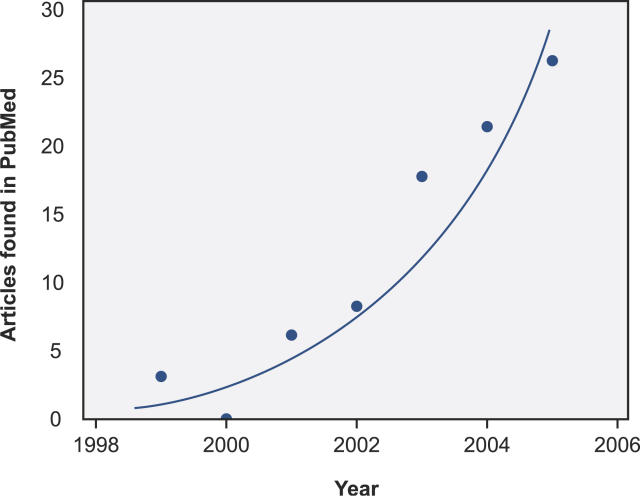
Estimation of Growth Curve for Immunomics Based on a PubMed Search See text for the search criteria used. (Illustration: Russell Howson)

While the growth boom in genomics took place in the 1990s and this field has now begun to enter a mature stage of development, a similar growth boom in immunomics is likely to take place over the remaining years of the current decade. We recently searched the PubMed database (with Sente 2.3) using the following query: “immunomics OR immunomic OR immunome OR (antigen AND microarray AND functional) OR (epitope AND microarray).” After removing nine irrelevant articles from the output list and adding five articles for the new journal *Immunome Research,* we obtained a list of 71 articles covering the years from 1999 to the present (please see Figure 1). It is clear that interest in this field has accelerated, supporting the expectation of a continuing boom in growth. It is expected that the number of publications will increase at an exponential pace as immunomic microarrays became commercially available for research use. As immunomic array technology evolves, we expect that immunomic arrays with a small number of features will eventually be designed for specific clinical diagnostic purposes and used regularly in medical practice. However, these clinical applications might still be in the distant future.

## Immunomic Microarray Technologies

The basic functioning principle behind all microarray technology is the binding, and subsequent measurement, of target biological specimens of interest to complementary probes arrayed in a spatially addressable fashion. Typically, a planar surface, such as a glass slide, is used to support an array of spots containing the probes. As a consequence of using spatially addressable probes, a large number of different targets can be measured in a single experiment. For example, in the case of DNA microarray technology, which provides the basic enabling technology for functional genomics, the targets are fluorescent mRNA molecules (indicators of genomic expression) that are hybridized to gene-specific DNA probes immobilized on a planar surface. In a similar fashion, the enabling technology for functional immunomics is the immunomic microarray. The basic technologies for immunomic microarrays that we consider in detail in this paper are antibody, peptide, and peptide–MHC microarrays (see Table 1 for a summary of these technologies). Other functional immunomic approaches include dissociable antibody microarrays [48], cell microarrays [49,50], serum microarrays [51], peptide libraries [52,53], and serological analysis of cDNA expression libraries (SEREX) [54–58]. There are significant technological challenges inherent in fabricating immunomic microarrays, including the identification of a workable surface coating for the glass, appropriate probe concentration and target incubation times, and suitable spot size and interdistance [59–64].

**Table 1:**
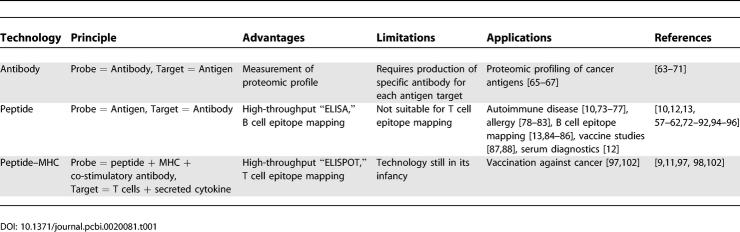
Summary of Basic Immunomic Microarray Technologies

Antibody microarrays consist of antibody probes and antigen targets; thus, they can be used to measure concentrations of antigens for which the antibody probes are specific [65,66]. As such, antibody microarrays are quite useful in *proteomic* applications, such as in the proteomic profiling of cancer antigens [67–69]. Antibody microarrays have also been proposed for post-translational functional genomics [70]. The rationale for directly measuring protein concentration, rather than using a traditional DNA microarray format, is the existence of evidence of poor correlation between concentrations of mRNA and its corresponding protein, which reflects post-translational modification of the protein [71]. Given the possibility of measuring antigens or proteins associated with “foreign agents,” antibody microarrays can be employed in functional immunomics applications [72,73] (The application in [73] actually used cells, which display the target protein markers on their surfaces.) As a general rule, however, using antibody microarrays in data-driven functional immunomic applications may be problematic. One of the main reasons is that this approach requires the production of specific antibody sets for use in defining each of the antigen targets, and the development of large numbers of interrogative features (antibody sets) is a tremendous challenge, since humoral responses are much broader than MHC-restricted T cell responses, are highly conformationally dependent, and can be developed against a great variety of chemical/biological elements present in biological fluids, including small molecules.

Peptide microarrays use the opposite technical approach; that is, they use antigen peptides as fixed probes and serum antibodies as targets [74]. This format is promising for functional immunomic applications. Published studies using peptide microarrays include applications to autoimmune disease [10,75–79], allergy [80–85], B cell epitope mapping [13,86–88], vaccine studies [89,90], detection assays [91,92], serum diagnostics [12], characterization of weak protein interactions [93], and analysis of antibody specificity [94]. Peptide microarrays essentially correspond to high-throughput parallelized ELISA assays [12,95–98] and thus can reveal the repertoire status of antigen-specific B cell antibody responses. However, B cell responses are highly dependent on CD4^+^ T cell immune responses, and thus peptide microarrays should ideally be used in parallel with extensive analysis of T cell responses (e.g., by using peptide–MHC microarrays; see below). One of the pioneer studies that best depict the usefulness of peptide immunomic technology was performed using an array of 87 protein antigens to search for specific antibody reactivity patterns in the serum of 20 normal health volunteers; these were compared to the patterns of 20 type-1 diabetes mellitus patients, and simple classifiers were designed to discriminate between healthy and diabetic patients, with an overall sensitivity of 95% and specificity of 90% [78]. In a subsequent study, the 87-feature array was able to identify prognostic signatures that could predict the susceptibility of healthy animals to develop diabetes [10]. Another important report describes the use of a panel of 225 selected peptides of several protein antigens known to be recognized by autoimmune disease patients [79]. The autoimmune peptide array was used to study the profile of the autoantibody reactivity pattern of rheumatoid arthritis (RA) patients. The RA study used serum from 18 RA patients, 38 healthy controls, and 58 recently diagnosed RA patients, and found early clinical prognostic markers able to predict which patients are more likely to develop severe RA, and also markers to identify the group of patients with the milder form of the disease [77]. Moreover, an array developed from a panel of 213 peptides derived from allergenic peanut proteins established that patients responding to a greater diversity of peptide peanut epitopes had the worst allergic reactions [80]. The importance of the breadth of antibody response against the simian–human immunodeficiency virus (SHIV), a experimental nonhuman primate model for human immunodeficiency virus (HIV), was demonstrated through studies performed with an array of 430 peptides derived from simian immunodeficiency virus (SIV) and HIV amino acid sequences [90]. This study indicated that the reduction of the repertoire of the antibody response was associated with development of acquired immunodeficiency syndrome (AIDS). These examples underscore the great potential of peptide microarrays to identify several valuable clinical markers.

The most recent technology to be proposed is the *peptide–MHC microarray* or artificial antigen–presenting chip [9,11,99]; in this case, recombinant peptide–MHC complexes and co-stimulatory molecules are immobilized on a surface, and populations of T cells are incubated with the microarrays, whose spots effectively act as artificial antigen–presenting cells [100] containing a defined MHC-restricted peptide. Different methods have been proposed for detecting T cells expressing receptors with affinity for specific peptide–MHC complexes on the microarray; these can include simple inspection of T cell clusters bound to a spot [9] or identification of activated cells secreting specific cytokines with cytokine-specific capture antibodies [11,99]. Peptide–MHC microarrays correspond to high-throughput parallelized ELISPOT assays [19], particularly when low enough densities of cells are used, in which case a direct counting of activated cells is possible [11]. Quantitation can involve cell counts alone, detected cytokine intensities alone, or a combination of both, as in [99], which used a cell-count score adjusted by an intensity score. The benefit of using peptide–MHC microarrays is that it can map MHC-restricted T cell epitopes, which are involved in several helper and regulatory functions of the immune system, and can be used in conjunction with peptide-based B cell epitope microarrays to study the adaptive system as whole.

**Figure 2:**
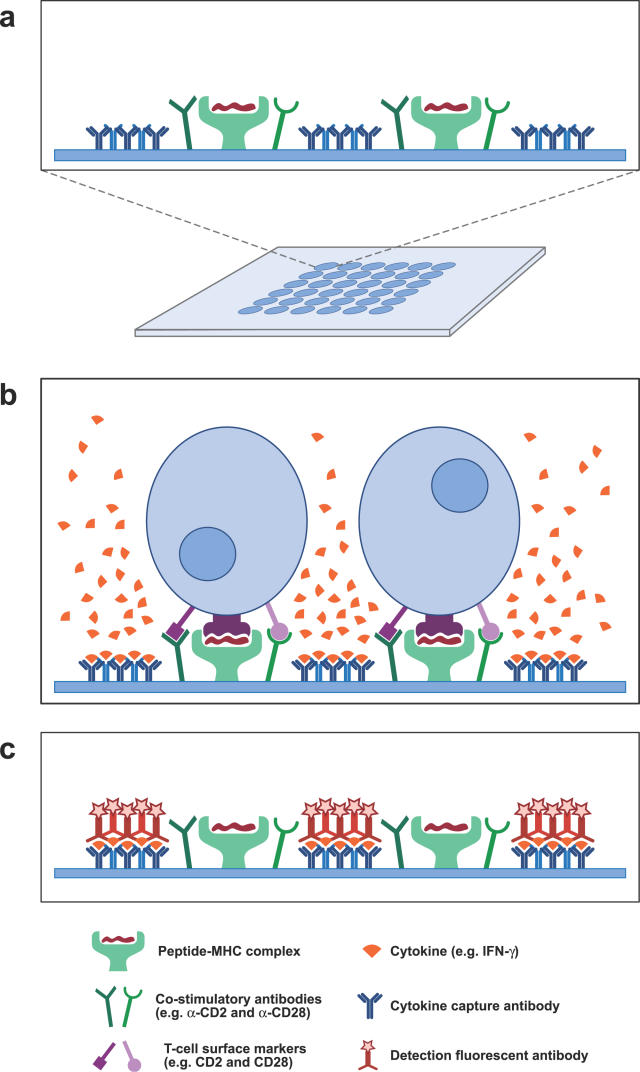
Peptide–MHC Immunomic Microarray Technology (a) Diagram of a peptide–MHC microarray, with an inset displaying a peptide–MHC spot, which includes co-stimulatory antibody needed to enhance T cell activation, as well as capture antibody to bind secreted cytokine. (b) Binding and activation of T cells on a specific peptide–MHC spot, which acts as an artificial antigen–presenting cell. (c) After washing, captured cytokine is revealed by the use of fluorescent antibodies, leading to a measurement of specific immunological response to the peptide–MHC complex. (Illusrtation: Russell Howson)

Figure 2 illustrates the functioning of peptide–MHC microarrays. In Figure 2a, a peptide–MHC microarray is depicted, with an inset showing the probe molecules that are deposited on a microarray spot. Figure 2b depicts T cells that bind to, and are activated by, specific peptide–MHC complexes, with the help of co-stimulatory antibodies; these T cells secrete cytokines that are captured by specific detection antibodies. Finally, as depicted in Figure 2c, the T cells and excess cytokine are washed away, and the bound cytokine is revealed by fluorescent antibody (other methods can be employed to reveal the cytokine [11]). Therefore, a peptide–MHC spot is designed taking into account two elements: a peptide–MHC complex, and the detection antibody specific to the particular cytokine one wants to measure. A third element can be the kind of T cell population (e.g., T helper or CTL) that is used as a target (i.e., incubated with the microarray). The choice among these three different elements will lead to a vast number of immunomic responses that can be measured.

**Figure 3:**
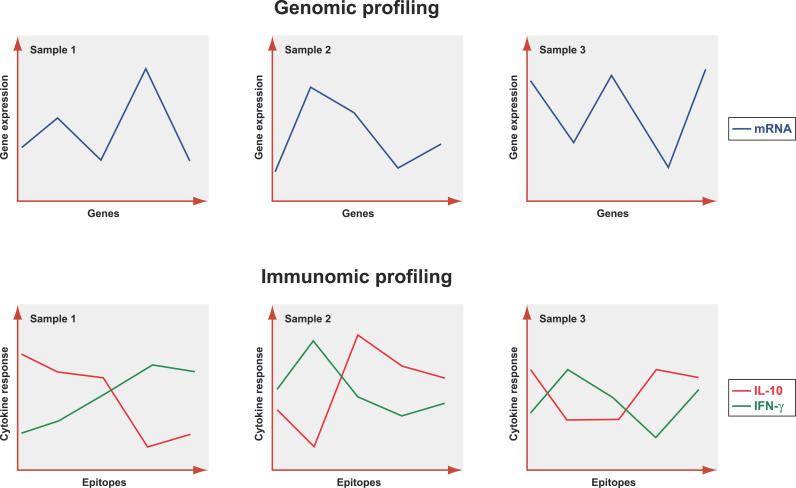
Genomic Profiling with DNA Microarrays Consists of One Signal (mRNA) Per Sample, while Immunomic Profiling with Peptide–MHC Microarrays Can Involve Multiple Signals per Sample For a given T cell population, one could measure for each epitope on the microarray the associated IFN-*γ* and IL-10 secretion, which corresponds respectively to inflammatory and anti-inflammatory activity, producing multispectral profiles. (Illustration: Russell Howson)

An exciting feature that distinguishes immunomic from DNA microarray data is the possibility of measuring two or more signals simultaneously, determined by a single feature, the epitope. In the case of DNA microarrays, one response value is obtained for each gene per sample, namely the concentration of mRNA produced by the gene (note that two-dye experiments use mRNA from two different samples). In the case of peptide–MHC chips, a single epitope can generate different response values corresponding to different cytokines or different target T cell populations, or even different antibody isotypes, in the case of peptide microarrays. In other words, in the case of genomic microarrays, one parameter is measured, namely the level of transcription of each individual gene, whereas in the case of immunomic microarrays, it is possible to measure several parameters regarding immune responses against a single epitope. For instance, a single B cell epitope can be recognized by different isotypes of immunoglobulins, such as IgE or IgG1. Therefore, in this case it is not only the intensity of the antibody response that can be measured, but also the quality of the antibody response. This aspect can be very relevant since a high IgE titer in relation to IgG1 may be associated with allergy, whereas the opposite, a high IgG1 titer in relation to IgE to the same epitope, is not. This situation is even more significant in the case of the peptide–MHC array, where the same peptide–MHC epitope can induce several different cytokine responses. These “multicolor” peptide–MHC microarrays have a counterpart in the multicolor ELISPOT assays currently in use [101]. It is known that the combined effect of multiple cytokines is essential to the control of immune responses; this is described by the suggestive term “cytokine chord” in [102]. Thus, given a family of epitopes, one may want to simultaneously measure both inflammatory (effector) and anti-inflammatory (regulatory) T cell responses, which are known to be associated with the concentrations of IFN-*γ* and IL-10, respectively [103]. In this case one would have more than one spot on the microarray containing the same epitope (peptide–MHC complex) but use distinct cytokine antibodies for detection (see Figure 3). The result of this analysis is not a real-valued profile, as is obtained from functional genomics microarrays, but rather a vector-valued profile. Such profiles are sometimes called “multispectral” profiles (see the section on data analysis below). We will, however, adopt the term “multicolor” when referring to immunomic data, due to the fact that this term is already used in the similar setting of ELISPOT assays.

The technological challenges mentioned previously in connection with antibody and peptide microarrays are much more complex in the case of peptide–MHC microarrays, which in fact involve elements of the two previous technologies, namely presentation of peptide and antibody detection of secreted cytokine. The technology of peptide–MHC microarrays, though still in its infancy, is viewed as a simple and economical method for screening the T cell repertoire of a host [99], and thus holds great potential. The first clinical research application of peptide–MHC microarray technology was the study of the correlates of protection regarding the effects of an experimental therapeutic cancer vaccine [104]. Ten patients with melanoma were immunized with a peptide vaccine, and their immune responses were examined with a peptide–MHC microarray, which contained seven types of peptide–MHC epitopes and probed for 26 secreted factors. This peptide–MHC array was shown to have the sensitivity to detect one peptide–MHC specific T cell in 10,000, and 10^6^ CD8^+^ cells were incubated with the array (so, in theory, this peptide–MHC array could detect up to 100 distinct reactive features that have reached minimum frequency of 1:10,000). Analysis of the peptide–MHC microarray response patterns demonstrated that patients who presented both IFN-*γ* and TNF-*α* secretory responses against a specific epitope remained free of melanoma.

## Computational and Statistical Challenges

The complexity of the statistical analysis with regard to immunomic microarray data is on a whole different level than that of genomic microarray data. The total number of genes in humans is estimated to be ~30,000; in comparison, the total number of different T cell receptors in humans, generated by somatic recombination, which recognize peptides within the context of a major histocompatibility molecule, is estimated to be on the order of 10^7^ to 10^15^, and the number of B cell clonotypes, generated by somatic recombination of V(D)J genes, is estimated to be on the order of 10^12^. DNA is based on a four-letter “alphabet,” consisting of the four nucleotide bases A,G,C,T, whereas peptide epitopes are based on a 20-letter alphabet, consisting of the amino acids known to be involved in life processes. Clearly, the combinatorial complexity in the case of functional immunomics is several orders of magnitude higher than that of functional genomics.

In addition, for functional genomics the number of interrogative features that need to be built on microarrays is on the order of 10^4^ to 10^5^. In functional immunomics, the total number of interrogative features included in microarray analysis can be much larger and may be estimated as follows, in the case of peptide–MHC microarrays: analysis of MHC peptide–binding motifs [26] suggests that a core of nine amino acids within a peptide is sufficient for characterization of a T cell epitope. The total number of interrogative features would thus correspond to the number of possible nine-letter words based on a 20-letter alphabet, which is 20^9^ ≈ 10^11^; fortunately, only <1% of these are able to bind to MHC molecules, which makes the number of interrogative features more manageable. In addition, B cells and T cells go through a process of clonal selection, where leukocytes that either do not react or react too strongly are eliminated, and dangerous clones that react with self antigens are deleted or anergized as part of the process of immune tolerance. The number of features is still very large, however, and methods to further reduce this number are essential; such methods include the prediction methods and immunomic databases mentioned previously in connection with epitope mapping, as well as the selection of peptides from specific genomes of pathogens, allergens, and self-antigens involved in human infirmities, such as tumor antigens, diabetes, and autoimmune diseases. One additional procedure that we and other groups have used after screening the genomes of pathogens for putative binding peptides is to compare those candidate epitopes with known host protein sequences, and in some cases we have found peptides that are identical to host peptides. This is very important because several critical diseases are caused by pathogen molecular mimicry, that is, some diseases, such as diabetes, dengue hemorrhagic fever, and Guillian Barret syndrome, are hypothesized to be the result of infections that induce self-reactive pathogenic immune responses.

It is important to state that the goal of the immunomic array is not to test all possible naïve T cell or B cell clones that can be generated by the somatic, VJ, or V(D)J recombinations against all possible combination of peptides—there simply would not be enough patient blood to do that, even if it were considered to be a relevant pursuit. The goal of the immunomic array is to identify primed cells that have reached a reasonable level of precursor frequency and are thus expected to have biological relevance. If the frequency of a circulating T cell in the peripheral blood is less than 1:100,000 we can expect that the biological relevance of it is small in contrast to a T cell that has a frequency of 1:1,000. In addition, a T cell has a binding affinity for the peptide–MHC that the T cell is specific for, and it should not bind to the ones with which it has no affinity. The current reported limit of detection of the peptide–MHC immunomic array is 1:10,000 cells when using 10^6^ CD8^+^ cells. So, in theory, a peptide–MHC array incubated with 10^6^ purified CD8^+^ cells (~10 ml blood) could detect up to 100 distinct reactive features that have reached minimum frequency of 1:10,000. However, if we expect to be able to detect rare clones, with very low frequencies, more cells would be needed. This requirement may be overcome with larger amounts of blood, and it is not unreasonable to collect 100 ml blood, or by adding a T cell expansion step to grow the cell population ex vivo before it is incubated with the array. This technique of T cell clonal amplification is commonly used to detect rare populations of cells by flow cytometry or by ELISPOT and may as well be applied in immunomic studies.

Normally, 250,000 human PBMCs are used in an ELISPOT assay with one peptide, and the limit of detection is estimated to be 4-fold to 5-fold more sensitive than flow cytometry. However, ELISPOT is quite distinct experimentally from immunomic microarrays. In ELISPOT, T cells and APCs are present in the same mixture, and for a T cell to be activated, it has to be in close contact with its APC. In the peptide–MHC array the spot surface is equivalent to a very large defined APC, completely loaded with one specific peptide epitope, for which a reactive T cell has a binding affinity, so that it adheres to its specific peptide–MHC spot and not to the other spots. In [104], the authors used 10^6^ cells and compared the limit of detection of the array with flow cytometry. It turned out that both had similar limits of sensitivity, approximately 1:10,000 cells or 0.01%. It is possible that in the future the peptide–MHC spot surface can be improved and the sensitivity of the peptide–MHC array may become even greater than the current ELISPOT assay.

Another great challenge is the polymorphism of HLA genes, in particular HLA class II, and the several combinations of different alpha and beta chains. Some approaches may be useful to limit the number of features, such as the selection of specific alleles most frequently found in a population to be used in broad screening arrays. A second such approach could be the use of supertype prototype HLA molecules compatible with a set of several HLA alleles. A third approach would be through customization of the arrays, by having many different arrays of single alleles and combining them according to the HLA types of the individuals being tested.

**Figure 4:**
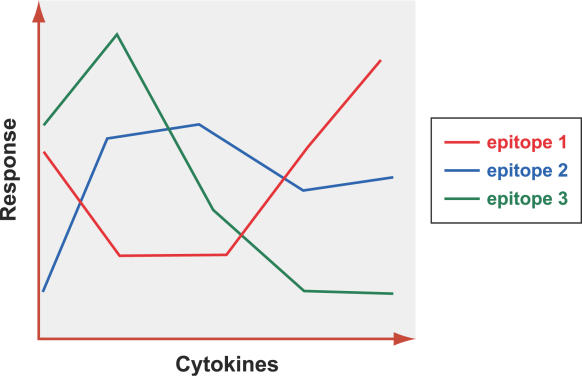
Spectral Signatures for Different Epitopes Associated with a Given T Cell Population Sample These are simply the measured secretion of different cytokines on microarray spots associated with the same specified epitope. (Illustration: Russell Howson)

An issue that sets immunomic microarray data apart is the availability of vector-valued response profiles (in the case of peptide–MHC microarrays). The statistical challenge here is reminiscent of the data analysis problem in the engineering field of remote sensing [105], where different materials have characteristic vector responses, called spectral signatures. In the case of immunomic data, the analogous notion to the spectral signature is the cytokine profile associated with a given epitope and T cell population; see Figure 4 for an illustration. One simple technique to address the data analysis problem for multicolor immunomic data is to combine the responses into one long feature vector, by juxtaposing the individual cytokine response profiles for each epitope, with the caveat that there may be systematic correlation among the features in the resulting feature vector.

The large number of features that can be measured simultaneously with microarray technology also presents a challenge. On one hand, it is likely that a large number of irrelevant features will be present; on the other hand, the scientist would like to work with a small number of strong, relevant features that can be used for diagnostic/prognostic panels, or as the basis for further biochemical validation studies of the mechanisms involved. This problem of feature selection also arises due to a fundamental limitation in statistics, sometimes called the “curse of dimensionality”, according to which the existence of a large number of features necessitates an even larger (exponentially larger) number of samples to achieve consistent and accurate results. As the number of patients in microarray-based studies is severely restricted by factors such as the cost of the technology and difficulties in patient enrollment, it is almost always the case that only a small number of samples are available. Thus, only a small number of features at a time can be considered. The recommended approach to feature selection is to consider combinations of *m* features at a time, do classifier design based on the feature set under consideration, and use an estimate of its probability of error as the performance score. Application of this type of analysis to immunomic microarray data would allow the identification of sets of epitope-specific immune responses, associated for example with distinct disease states. However, feature selection presents an explosive combinatorial problem. For example, in an exhaustive selection of sets of three features among 1,000 initial features, the total number of feature sets to be assessed is equal to 166,167,000. If an initial set of 10,000 features is used instead, the number of feature sets of size three to be searched is larger than 10^11^. The complexity of feature selection is especially crucial in functional immunomics applications, since in this case the number of initial features to be considered is huge. The use of high-performance computing architectures, such as large computer clusters, is almost mandatory.

**Figure 5:**
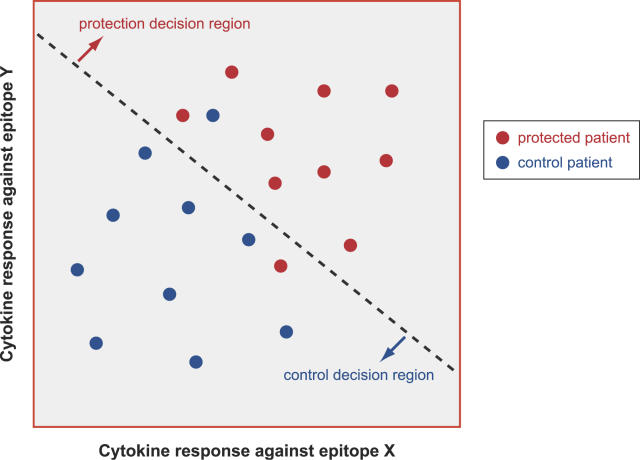
Example of a Linear Classifier The response to epitopes X and Y discriminates the patients protected by immunization from the control patients. (Illustration: Russell Howson)

Given that a set of features has been selected, one should be able to design a classifier that takes as input microarray data for an unknown sample and generates as output a predicted class label (e.g., clinical outcome, kind of infection, or other conditions). Figure 5 illustrates this approach in a hypothetical functional immunomics application. In this case, there are two class labels, corresponding to control and protected patients, in a situation in which protection is achieved by immunization with an attenuated-virus vaccine for a given infectious disease. The objective is to identify the epitopes that show a discriminatory response between the two groups and are therefore prime targets for rational epitope-based vaccine design. A set of two features, corresponding to epitopes X and Y, have been identified via feature selection among the thousands of microarray probes. For instance, we can imagine a situation in which the protected individuals presented a higher TNF-*α* response to epitope X, as well as a higher IFN-*γ* response to epitope Y. Based on the response values observed for each patient (note that a patient corresponds to a point in the plane), a linear classifier is designed. This classifier corresponds simply to two decision regions separated by a line. If a future unknown patient has response values that fall in the upper decision region, he/she is likely to be a protected patient, provided that the classifier has a small probability of error. In this case, the responses to epitopes X and Y (in terms of the TNF-*α* and IFN-*γ* cytokines) characterize immunological memory induced by the attenuated-virus vaccine: large response values to both epitopes X and Y indicate protected patients (note that the response of neither epitope X nor epitope Y by itself is a good discriminator in this example, indicating the need to consider the multivariate, combined effect of both responses). Note, in Figure 5, that the apparent error rate (i.e., the number of misclassified sample divided by the total number of samples) is 2 ÷ 20 = 10%; the actual probability of classification error on future data typically exceeds the apparent error rate [106].

## Systems Biology and Computational Knowledge Discovery: Immunomic Regulatory Networks

Systems biology makes use of mathematical modeling in order to provide a theoretical core for biology, analogous to the way that mathematical theories provided that core for physics in the 20th century. The success of engineering and computational methodology in the physical realm is due to the predictive capability of mathematical modeling. We quote from [107]: “Predictive mathematical models are necessary to move biology in the direction of a predictive science. They are also necessary to the application of engineering methods to translate biological knowledge into therapies with a mathematical and computational basis.” The large complexity of biological systems, in comparison with most physical systems, makes even more urgent the application of mathematical and computational modeling techniques to them. Dynamical systems provide “the natural language needed to describe the ‘integrated behavior' of systems coordinating the actions of many elements” [108], and are also capable of displaying emerging self-order from massively disorganized complexity, which is believed to be a fundamental feature of life. In what follows, we will describe the notion of immunomic regulatory networks, a dynamical system model for immune regulation.

An important recent development in immunology has been the discovery of regulatory T cells [103,109–111]. These T cells suppress immune responses, helping to stem runaway inflammatory processes and to avoid autoimmune disease. It is thought that this beneficial suppression activity can turn deleterious when it is taken advantage of by pathogens, leading to chronic and abnormal infectious processes. It has been observed that some regulatory T cells are not antigen-specific; these are called natural regulatory T cells [103]. In addition, there exist regulatory T cells, both CD4^+^ and CD8^+^, that are antigen-specific and thus epitope-driven [103]. Given the suppressive action of epitope-driven regulatory T cells in conjunction with the promoting activity of epitope-driven helper T cells, it follows that the immunological response to a given epitope may be suppressed or promoted by the immunological response to other epitopes. Thus the notion of regulatory networks arises as a fundamental concept in understanding the functioning of the immune system. In fact, most human diseases are the result of an unbalance in immune system homeostasis.

In functional genomics, DNA microarray data is used to infer genomic regulatory networks [112]. For biological and efficiency reasons, gene expression is often quantized to two levels: on and off [113]. The multivariate methods of classification and feature selection discussed in the previous section have proved to be essential in the inference of such Boolean (binary) regulatory networks. By the same token, immunomic microarray data can be used to infer immunomic regulatory networks. As is true for gene expression, one may quantize each epitope response measured with an immunomic microarray at one of two levels—on (immunogenic/responder) and off (nonimmunogenic/nonresponder)—leading to the inference of Boolean immunomic regulatory networks.

**Figure 6:**
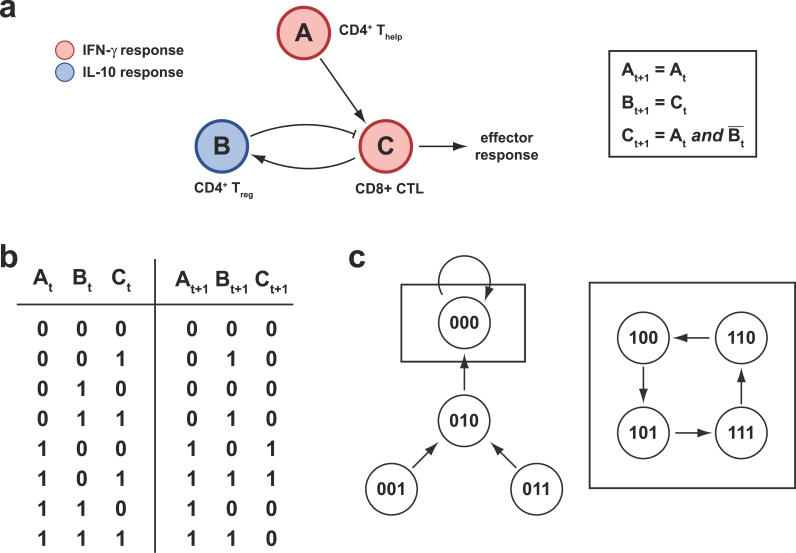
Example of a Simple Immunomic Network, Consisting of Three Epitopes Epitope A is a promoter (A is specific to CD4^+^ helper T cells), epitope B is a suppressor (B is specific to CD4^+^ regulatory T cells), while epitope C produces the effector response (C is specific to CD8^+^ cytotoxic T cells), while also promoting the suppressing response of epitope B (negative feedback). (a) Network wiring diagram and transition rules. (b) State transition table. (c) Basins of attraction in state-space, with attractors indicated by dashed rectangles. (Illustration: Russell Howson)

In the general case, each node of an immunomic regulatory network represents a combination of the epitope, the cytokine response measured, and the T cell population used as the target; in most cases, each node is in a one-to-one relationship with a single physical spot on an immunomic microarray experiment with a given T cell population. The edges between nodes represent putative regulatory relationships between the cells that respond to the respective epitopes. Figure 6 depicts a simple example with quantized Boolean responses, where peptide–MHC microarrays are used in conjunction with three kinds of T cell targets: CD4^+^ helper T cells, CD4^+^ regulatory T cells, and CD8^+^ cytotoxic T cells. Epitope A is specific to the CD4^+^ helper T cells that promote the response to epitope C, which is specific to the CD8^+^ effector T cells that produce the actual protective mechanism. In addition, there is an epitope B that activates the CD4^+^ regulatory T cells that suppress the effector response to epitope C, thereby producing an anti-inflammatory response. In practice, such a model would be derived from microarray data by automatic epitope (feature) selection and determination of predictive relationships (classifier design). In this example, the effector response is activated only in the presence of both help from epitope A *and* an absence of regulatory response to epitope B. The suppressing response to epitope B is itself promoted by the presence of a response to epitope C, providing a negative feedback mechanism. These relationships can be represented by a wiring diagram and transition rules, depicted in Figure 6a.

Since the responses have been quantized to two values, on and off, and there are three epitopes, the total number of possible states of the system is 2^3^ = 8. Figure 6b depicts the state transition table obtained from the transition rules in Figure 6a. Using this table, one can determine the attractors of the system, which are the states or sets of states in which the system stays in the long run in the absence of external disruptions. For each attractor, there is associated a basin of attraction, containing attractors and transient states, which are the sets of states that lead to the attractor [108]. In the context of biological systems, attractors are a mathematical model for homeostasis. In our minimalist example, we see that there are two different behaviors, corresponding to the two distinct basins of attraction in Figure 6c; the respective attractors are indicated by dashed rectangles. As can be seen, which of the two behaviors the system is in depends only upon the state of the response to epitope A. If it is off (there is no help), then the system may pass through some irrelevant transient states but it will always tend toward the resting single-state 000 attractor and thus an absence of activity; see the diagram on the left in Figure 6c. If the response to epitope A is on (there is help), then the activity of the system corresponds to that of a cyclic attractor, with the effector response being turned on and off cyclically; see the diagram on the right in Figure 6c. This situation corresponds to modulation of the effector response and regulation of the inflammatory response by means of a negative feedback mechanism. Note that when there ceases to be a response to epitope A, the system jumps to the other basin of attraction, and tends to the resting 000 state. The immune response to epitope A in this example determines the behavior of the system, and thus it functions as the master of the overall immunological response, with the individual immune responses to epitopes B and C being slave to it. The concept of master–slave regulatory units is quite important for the understanding of complex regulatory systems and has in fact been considered as a mechanism for genomic regulation (Michael Bittner, unpublished data).

Inference of immunomic regulatory networks from immunomic microarray data constitutes, after proper validation, computational knowledge discovery. There are subtle epistemological issues involved in using data-driven, computer-based methodology to obtain scientific knowledge. As Karl Popper explained in his classic book *The Logic of Scientific Discovery* [114], every scientific theory consists of an initial irrational act of creativity (induction) followed by rigorous logical consequences (deduction) and testing of the initial hypothesis. Where is the initial act of creativity, in other words the scientific hypothesis, in computational knowledge discovery? Computers are clearly not capable of irrational creativity. Can this be considered science? We maintain that the answer is yes [115]. In fact, the initial irrational act of creativity is there; it is involved in all the steps of experiment design, selection of patients/samples, and choice of statistical methodology. Once these are settled, the actual data analysis *is* purely logic deduction via the machinery of mathematical operations, as prescribed by Popper. In this deductive stage the computer plays a critical role, as it facilitates the application of very complex computational methods. Therefore, scientist (and statistician) bias here is in fact unavoidable, as it is in all scientific disciplines. In particular, the term *data-driven* is really a misnomer in describing computational knowledge discovery.

## Conclusion

Functional immunomics promises great rewards, both in terms of our basic understanding of the immune system and in disease diagnosis/prognosis and rational epitope-driven vaccine design. Research into the basic biology and statistical methods associated with functional immunomic experiments will lead to the advancement of medical science and public health. Functional immunomics is however still at an early stage of development. In this review we have attempted to provide a coherent vision of this nascent field, and have speculated on future research directions for this technology.

In our discussion we have often compared and contrasted immunomics with genomics. Immunomics supervenes on genomics, in the epistemological sense, since immunology ultimately depends on the functioning of genes inside cells, but immunomics has its own independent character and properties. In the same manner that each cell has its own pattern of gene expression that defines its unique cellular properties, each reaction of the cognate immune system to an antigen has its own pattern of epitope-specific responses that define its final outcome.

There exists a large collection of mathematical models to describe the immune system [116]. Here, we have proposed Boolean immunomic regulatory networks as a new mathematical model for immune system regulation. This is a dynamical system model, with parameters that can be estimated from immunomic microarray data. A somewhat similar concept was suggested in [102,117], where a network model for cytokine action was proposed, albeit without explicit reference to large-scale immunomic technology or regulatory T cell response. In addition, immune system regulatory networks have been previously discussed in the context of genomics [118]. The immunomic regulatory network model may be useful for computational knowledge discovery and simulation of regulatory mechanisms of the immune system in health and disease, which may lead to advances in practical applications (e.g., vaccine design) as well as in the basic scientific understanding of the immune system.

## Abbreviations

RA: rheumatoid arthritis
